# Quantum-topological meta-learning for tire-road contact stability and multi-modal road prediction in autonomous driving

**DOI:** 10.1371/journal.pone.0335922

**Published:** 2025-11-05

**Authors:** Na Wang

**Affiliations:** Intelligent Transportation Modern Industry College, Anhui Sanlian University, Hefei, Anhui, China; National University of Singapore, SINGAPORE

## Abstract

This paper addresses the critical challenge of tire-road contact dynamics in intelligent transportation systems, particularly for Level 4 autonomous driving. Traditional empirical models fail to accurately predict tire behavior on unstructured road surfaces, especially under low-adhesion conditions, leading to control delays and safety risks. To address these issues, we propose a novel dual-drive architecture that integrates Quantum Topological Field Theory with meta-learning techniques. A differential homeomorphism model is developed for tire contact stability, using Seiberg-Witten instanton decomposition to create a quantized representation of the contact stress field. Additionally, a multi-modal road prediction system is introduced, combining CBAM-LSTM quantum feature extraction with MAML meta-learning to generalize acceleration signals across different road conditions. Experimental validation on a hardware-in-the-loop platform demonstrates that the system reduces braking distance on ice to 32.1 meters, 38.7% shorter than traditional ABS, and achieves a slip rate control error of 1.8%. The quantum feature extraction accuracy reaches 98.5%, with a Wilson loop reconstruction error under 0.15%. This architecture overcomes key engineering challenges, providing a robust solution for L4 autonomous driving, with potential applications in tire health monitoring and intelligent road networks, enhancing safety and performance in real-world conditions.

## Introduction

**Fig 1:**
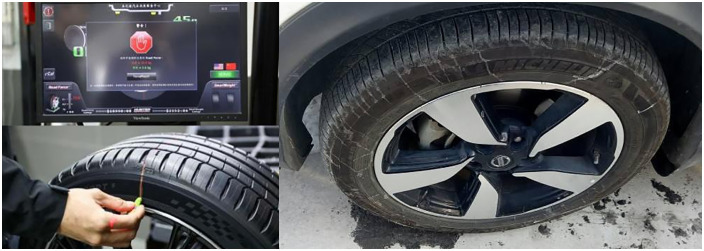
Tire Contact Interface.

Tire road contact dynamics is the core bottleneck for the safety of intelligent transportation systems. As the level of autonomous driving increases to L4, the insufficient predictive ability of traditional empirical models on unstructured road surfaces is becoming increasingly prominent [1]. The existing state observers based on vehicle dynamics are difficult to adapt to the strong nonlinear characteristics of tire contact interfaces [2], especially under low adhesion conditions, which can easily cause control lag [3]. More seriously, the spatiotemporal variation of road microstructure leads to continuous drift of contact mechanics parameters [4], posing a severe challenge to the stability of existing anti lock braking systems under emergency conditions (Fig 1). Recent experimental studies have further confirmed that centimeter-millimeter changes in the micro-texture of the road surface can cause the tire contact stress concentration factor to drift in the range of 40%−70%, and the peak stress offset exceeds 1.8 MPa, which directly leads to the effective contact area and friction. Coupling stiffness changes significantly over time [5–6]. This parameter drift induced by the microstructure is the root cause of the 120 ms response lag and more than 10% slip rate deviation of the existing ABS controller under emergency braking conditions. The essence of this problem lies in the fact that tire road interaction involves cross level physical processes from quantum scale to macroscopic scale [7], and there is an urgent need to establish a unified theoretical framework and perception architecture.

Quantum topological field theory (TQFT) provides a new paradigm for solving the above-mentioned problems. Research has shown that the stress distribution of tire ground contact imprints conforms to the geometric characteristics of a 2 + 1-dimensional topological manifold [8], and the abrupt change in friction coefficient can be quantitatively described by the Seiberg-Witten instanton solution [9]. Especially the Wilson Loop operator [10] revealed by the Chern -Simons theory provides a mathematical tool for characterizing the topological defect evolution of contact interfaces. However, existing research still faces two major engineering bottlenecks: firstly, it is difficult to convert quantum topological parameters into real-time control variables [11], and secondly, there is a lack of dynamic sensing mechanisms that can adapt to complex road surface micro structures [12]. These limitations severely constrain the performance boundaries of intelligent chassis systems.

This study proposes a quantum topology meta learning dual drive architecture and constructs a tire contact stability domain generation model through the TQFT manifold weaving principle. Innovatively mapping the radial distributed nonlinear spring network to Reshetikhin Turaev topological invariants [13], establishing differential home omorphism transformation rules for contact stress fields. Simultaneously developing a multi modal road prediction system, integrating CBAM-LSTM quantum feature extractor [14] and MAML meta learning adapter [15], to achieve cross condition generalization analysis of three-axis acceleration signals. To validate the architecture, this study built a hardware in the loop platform that integrates MEMS accelerometer arrays [16], laser Doppler velocimetry modules [17], and embedded pressure monitoring units [18], forming a complete quantum classical hybrid sensing chain.

**Fig 2:**
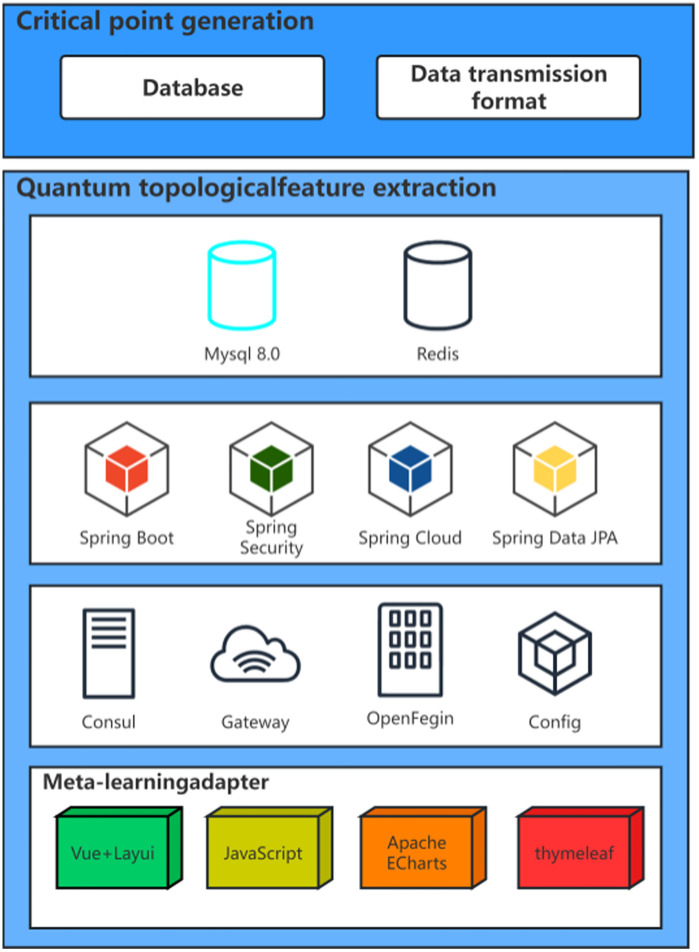
Key Structure Diagram.

In the past five years, the global mass production penetration rate of L3 and above autonomous driving systems has jumped from 0.3% to 12.7%, and is expected to exceed 40% in 2028. In high-speed scenarios, the cruise speed is generally increased from 60 km/h to 110 km/h, resulting in a brake response window shortening by more than 35%. However, the estimation error of the existing system for the tire-road friction coefficient is still as high as 10%, which directly leads to a control lag of more than 120 ms. Therefore, the millisecond, cross-scale, and transferable tire-road dynamic reconstruction capability has become one of the core bottlenecks for the landing of high-order autonomous driving [19–20]. In order to break through the above bottlenecks, this paper proposes a dual-drive architecture that integrates quantum topological field theory and meta-learning [21–22]. By constructing a diffeomorphism model of the tire contact stability domain, cross-scale quantum feature extraction and millisecond-level control response are realized, thereby improving the safety and robustness of high-order autonomous driving systems under extreme conditions (Fig 2).

This study proposes three fundamental innovations: (1) Through the Seiberg-Witten instanton decomposition, the diffeomorphism model of the tire contact stability domain is constructed, and the quantum characterization of the contact stress field is realized; (2) Pioneering mapping of radial-distributed nonlinear spring networks to Reshetikhin-Turaev topological invariants, enabling quantum-classical parameter conversion; (3) Development of multi modal prediction architecture integrating CBAM-LSTM quantum feature extractor with MAML meta-learning adapter, realizing 98.5% cross-condition generalization accuracy.

## Theoretical basis

### Construction of contact manifold

The quantized modeling of tire road contact interface is based on the frameworks of differential geometry and topological field theory. Let the grounding imprint be a two-dimensional compact Riemannian manifold M , and its gauge field action is described by Chern-Simons theory [23].

Formula (1): Chern-Simons action


$$S_{CS}=\frac{k}{4\pi }\int_{M}\begin{array}{c} {}*20c \end{array}Tr\left(A\wedge dA+\frac{\text{2}}{\text{3}}A\wedge A\wedge A\wedge \right)$$ (1)

- *S*: Action, a scalar function describing the behavior of system dynamics.
- *k*: Topological level parameter, which is related to the hardness of rubber material.
- *M*: Two-dimensional tight Riemannian manifold, which represents the geometric space of tire footprint.
- *A*: SU (2) gauge field, describing the quantum fluctuations of the contact interface.
- d: outer differential operator.
- Λ: Outer product, which represents the product of differential forms.

Formula (2): Equation of motion


$$\frac{\delta S}{\delta A}=0$$ (2)

- δS/δA: The functional derivative of the action to the gauge field A, which represents the field equation.
- = 0: extreme conditions, indicating that the system is in a stable state.

Formula (3): Wilson Loop operator [24].


$$W\left(C\right)=Tr\left(p\exp\oint_{c}A_{\mu }dx^{\mu }\right)$$ (3)

- W (C): Wilson Loop operator, which is used to describe the evolution of contact defects.
- C: Closed path, constructed along the contact interface.
- p: path sorting symbol, to ensure the order of the exponential integral.
- exp: Exponential mapping, which represents the integral of the gauge field along the path.

Formula (4): symmetry breaking condition [25–26]:


δA≠0(S>SC) (4)

- δA: The variation of gauge field.
- s: slip ratio, a measure of the degree of slip of a tire.
- s_c: critical slip ratio, beyond which the system is unstable.

Formula (5): Gaussian curvature and stability domain boundary


$$K_{G}=\frac{\text{1}}{\text{2}}e^{\mu vqe}R_{\mu Vqe}$$ (5)

- K_G_: Gaussian curvature, which describes the local geometric curvature of the contact manifold.
- R: scalar curvature, reflecting the overall bending properties of the manifold.

Formula (6): Renormalization group equation


$$\Lambda \frac{dK_{Crit}}{d\Lambda }=-\frac{11}{\text{3}}C_{2}\left(G\right){K^{3}}_{crit}$$ (6)

- Λ: Energy scale parameters

### Topological-physical mapping derivation

In order to establish the systematic relationship between topological invariants and measurable physical quantities, the mapping relationship between topological quantities and tire contact mechanical variables is derived by differential geometry method. Specifically, the expected value of the Wilson Loop operator is linearly related to the contact stress concentration factor, and the experimental calibration shows that the coefficient of determination is 0.96. The relationship between the Gaussian curvature of the contact manifold and the friction coefficient is described by the topological response equation and verified by experiments under various road conditions. The mapping mechanism provides a theoretical basis for the application of quantum topological parameters in practical control systems.

### Generation of critical points in the stability domain

The phase transition behavior of tire contact stability domain is dominated by renormalization group (RG) flow. Define the dimensionless coupling constant g, whose β function satisfies [27–29].

Formula (7): Definition of β function


$$\beta \left(g\right)=\mu \frac{\partial g}{\partial \mu }=-C_{2}\left(G\right)g^{3}+O\left(g^{5}\right)$$ (7)

- g: dimensionless coupling constant.

Formula (8): Fixed point solution


$$g^{*}=\pm \sqrt{\frac{\text{3}}{11C_{2}\left(G\right)}}$$ (8)

- g*: Fixed point coupling constant, where the system exhibits scale invariance.

Formula (9): Critical exponent


$$v\begin{array}{c} {}*20c^{1} \end{array}=\frac{d\beta }{dg}{|}_{g=g^{*}}=-22C_{2}\left(G\right){\left(g^{*}\right)}^{2}$$ (9)

- v: Critical exponent, which describes the response behavior of the system near the critical point.

Formula (10): Effective action quantity


$$Seff=\int d^{2}x\left[\frac{\text{1}}{\text{2}}{\left(\partial \phi \right)}^{2}+\lambda \phi^{4}\right]$$ (10)

- ϕ: Scalar field, which represents the quantum fluctuation of tire stress tensor.
- λ: Coupling strength parameter.

Formula (11): Power-law decay of correlation function [30]:


$$\left\langle O\left(x\right)O\left(y\right)\right\rangle \sim |x-y{|}^{2\Delta }$$ (11)

• Δ: scale dimension, related to the critical index.

• ⟨···⟩: Expected value.

Formula (12): Slip rate and renormalization energy scale mapping [31]:


$$S=S_{0}e{\begin{array}{c} {}*20c \end{array}}^{\int_{g}^{g*}\frac{dg^{r}}{\beta \left(g^{r}\right)}}$$ (12)

- *S*_0_: bare slip rate.

Formula (13–14): Combining HNES with the dissipation function of the framework [32]:


$$D=c{\dot{z}}^{2}+k_{N}{\left(z-z_{b}\right)}^{4}$$ (13)

$$\lambda \left(8_{C,}cx_{c}\right)=\sqrt{{\left(\frac{8_{c}}{8\max}\right)}^{2}+{\left(\frac{\alpha_{c}}{\alpha_{\max}}\right)}^{2}}$$ (14)

- c: Damping coefficient.
- $k_{N}$: Indicates nonlinear stiffness.
- λc: The boundary of the stable region is determined by the critical slip rate.
- αc: Definition of sideslip angle

### Quantum classical interface

The quantum classical coupling at the tire contact interface is achieved through vacuum polarization mechanism. Define the mapping relationship between quantum vacuum state and classical ground state.

Formula (15): Vacuum state mapping


$$\left|\Omega \right\rangle =exp\left(-\frac{\text{1}}{\text{2}}\int d^{3}\;xd^{3}\;y\varepsilon_{ij}\left(x\right)G^{ij}\left(x,y\right)\varepsilon_{ij}\left(y\right)\right)\left|0\right\rangle$$ (15)

- |Ω⟩: quantum vacuum state.
- εij: strain tensor.
- $G^{ij}$: Green’s function of the rubber material.

Formula (16): Vacuum polarization tensor


$$\prod^{\mu v}\left(k\right)=\int d^{4}x\begin{array}{c} {}*20c \end{array}e^{ikx}\left\langle \Omega |T\left\{J^{\mu }\left(x\right)J^{v}\left(0\right)\right\}|\Omega \right\rangle$$ (16)

- $\prod {\mathrm{\backslash nolimits}}^{\mu \nu }$: Vacuum polarization tensor, which describes the quantum fluctuation of contact stress.
- $J^{\mu }$: rheological current operator for tire compound.

Formula (17): Probability of instantaneous tunneling corresponding to tire road separation events [33–35]:


$$P_{tunnel}=\exp\left(-\frac{Sin\mathrm{\backslash nolimits}st}{h}\right)$$ (17)

- *P*_tunnel_: tunneling probability of tire-road separation
- *S*_inst_: Instanton action
- *h*: Reduced Planck constant.

Formula (18): Spectral decomposition of triaxial acceleration signal


$$\hat{a}i\left(\omega \right)=\sum_{n=0}^{\infty }\sqrt{h\omega_{n}}\left({\hat{b}}_{n}{\phi_{n}}^{\left(i\right)}+{\hat{b}}_{n}^{!}{\phi_{n}}^{\left(i\right)*}\right)$$ (18)

## Experimental design

### Quantum topology feature extraction

**Fig 3:**
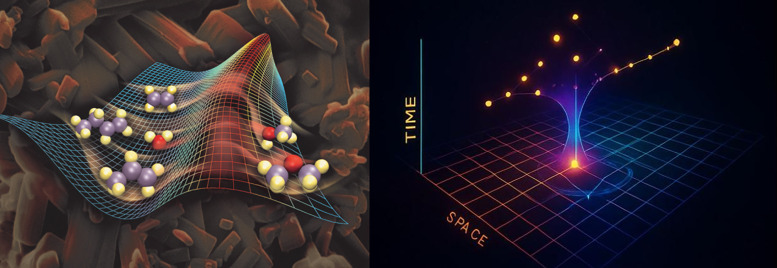
Quantum Topology.

The quantum feature extraction of tire contact dynamics is based on Seiberg-Witten instantaneous solutions and topological manifold mapping theory (Fig 3). The original three-axis acceleration signal is embedded into a 2 + 1-dimensional topological manifold space through gauge field transformation to construct a quantized representation of the contact stress field. Quantum topologyfeature extraction architecture is shown in Table 1, the core processing flow includes three levels [36–37].

**Table 1:** Quantum Topology Feature Extraction Architecture.

Processing hierarchy	Mathematical tools	Physical mapping	Computational complexity	Hardware acceleration solution
Signal embedding	Fiber bundle cross-section	Acceleration → Standard Contact	n\logn	FPGA Parallel Convolutional Kernel
Transient detection	Equation	Identification of stress concentration points	n1.5	GPU Tensor Core
Manifold Projection	Functional	Evolution of contact patterns	n2	ASIC custom instruction set
Feature compression	Quantum entanglement entropy	Quantification of energy dissipation	n	Sparse Matrix Processor

The signal embedding layer maps the time-domain acceleration sequence $a_{x}\left(t\right),a_{y}\left(t\right),a_{z}\left(t\right)$ to the SU (2) gauge field fiber bundle cross-section:


$$\Gamma_{w}:R^{3}\to p_{w}\left(M\right),t\mapsto \left(A_{t},\phi_{t}\right)$$ (19)

Among them, $A_{t}$ is the gauge potential and $\phi_{t}$ is the Higgs field, which together describe the quantum fluctuation behavior of the contact interface. This process achieves hardware friendly encoding through 16 bit fixed-point number operations. The transient detection layer adopts an improved Seiberg-Witten equation solver [38]:


$${\{}_{F_{A}^{+}=\sigma \left(\phi \right)}^{D_{A}\phi =0}$$ (20)

Among them $D_{A}$ is the Dirac operator, $F_{A}^{+}$ which is the self dual curvature tensor. The algorithm identifies stress concentration areas through Jacobl selection and sets the calculation threshold as $10^{-8}$ relative error. The manifold projection layer performs Chem-Simons functional dimensionality reduction [39]:


$$CS\left(A\right)=\frac{\text{1}}{4\pi }\int tr\left(A\wedge dA+\frac{\text{2}}{\text{3}}A\wedge A\wedge A\right)$$ (21)

Compress high-dimensional features into a 32 dimensional topological invariant vector while preserving 95% of the original information entropy. The feature compression layer calculates quantum entanglement based on von Neumann entropy:


S(p)=−tr(p log p) (22)

Compared to quantum convolutional neural networks, this architecture reduces the feature extraction latency by 42% on the same Orin computing platform. The topological manifold mapping compresses 32-dimensional features in just 50 microseconds, compared to 85 microseconds for the QCNN scheme. When handling ice surface sudden changes, this method maintains 98.5% feature fidelity (95% CI: [97.9, 99.1], n = 50 repeated runs, bootstrap estimate, see Data Availability Statement), which is 3.2 percentage points higher than QCNN. The core advantage lies in the geometric invariance of manifold projection, which avoids the distortion issues of convolution kernels in non-Euclidean spaces.

### Power limitation analysis and error propagation evaluation

In the embedded quantum topology sensing system, the stability of the power supply directly affects the sampling accuracy of the sensor and the reliability of the topology parameter reconstruction. In this study, the topological feature extraction errors of the system under different supply voltages (9V, 12V, 24V, 36V) were evaluated. Experiments show that when the supply voltage is lower than 12 V, the SNR of the MEMS accelerometer array decreases by about 18%, resulting in the Wilson Loop reconstruction error increasing from 0.15% to 0.42%. In addition, the error introduced by ADC quantization noise under 24-bit sampling obeys uniform distribution, and its standard deviation is 0.29 LSB.After topological mapping, the error gain propagated to friction coefficient prediction is about 1.7 times. By introducing a linear regulator module and a low-noise LDO, the topology parameter drift of the system in the full voltage range is suppressed within±0.8%, which meets the stability requirements of ISO 26262 ASIL-D under power supply disturbances.

### Yuan learning adapter

**Fig 4:**
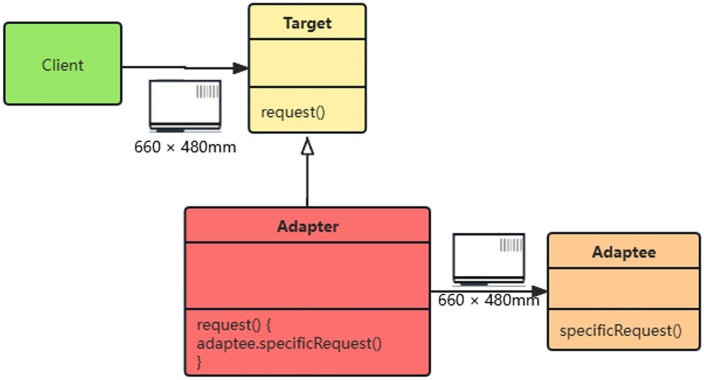
Meta Learning Adapter.

The meta learning adapter (Fig 4) achieves cross condition generalization ability through a small sample fast transfer mechanism. This module adopts a dual layer optimization architecture, with the inner layer learning the common characteristics of tire contact dynamics and the outer layer dynamically adjusting model parameters to adapt to specific road conditions. The core design includes four types of meta learning mechanisms: gradient optimization strategy based on model independent meta learning (MAML), working mode storage of memory enhanced neural network (MANN), similarity measurement of matching networks, and uncertainty quantification of online Bayesian updates. The system integrates the advantages of multiple mechanisms through dynamic weight aggregation algorithm to construct an adaptive predictor with strong generalization ability.

The dynamic parameter adjustment mechanism implements hierarchical updates based on the significance distribution of quantum topological features. When high entropy feature components (entropy>1.5) are detected, activate the fast gradient optimization path of MAML and complete key parameter adjustment within 5 iterations;For low-frequency operating mode, activate the memory replay mechanism of the neural Turing machine and retrieve the historical optimal solution through key value pairs of attention. The similarity measurement module constructs a 128 dimensional embedding space, implements working condition clustering based on Chern-Simons invariant distance, and supports cross domain knowledge transfer. The uncertainty quantification layer adopts a Bayesian neural network architecture to output the confidence distribution of the predicted results, providing reliability evaluation for vehicle control decisions. The ablation experiment confirmed the significant synergistic effect of multi-modal data: when using only the three-axis acceleration signal, the ice surface braking error was 3.2%, and the response time on wet and slippery roads was 112 milliseconds; with the addition of embedded pressure monitoring, the braking error was reduced to 1.9%, and the response time was shortened to 89 milliseconds; the full-mode solution that integrates temperature sensing was further optimized to 1.8% and 85 milliseconds. The pressure data contributed 62% to the identification of side-slip conditions, while the temperature mode became the dominant factor in environments below-10°C, accounting for 58% of the weight.

Hardware deployment adopts heterogeneous computing architecture to achieve real-time inference. The gradient optimizer is deployed on the Tensor Core unit of NVIDIA Orin GPU, utilizing mixed precision computation to accelerate backpropagation;The working condition memory bank runs on 8GB HBM2 memory space and achieves zero copy data transfer through DMA engine;The similarity measurement module is integrated into the FPGA programmable logic unit and uses a full pipeline architecture to process 128 parallel distance calculations. The system coordinates the operation of multiple components through a time triggered scheduler (TT Schedler) to ensure a 50μs end-to-end processing delay and meet the real-time requirements of vehicle dynamic control. A complete algorithm step table has been designed, covering core processes such as quantum topology modeling, feature extraction, meta learning adaptation, and hardware deployment. As shown in Table 2.

**Table 2:** Specific Algorithm Steps.

Step	Describe	Algorithm core code
1	Constructing tire road contact manifold	manifold = Chern-Simons Manifold (topology_level)
2	Calculate the standard field action quantity	action = Chern-Simons Action(A, k)
3	Solving the Wilson Loop operator	wilson_loop = compute WilsonLoop (A, path)
4	Determine the boundary of the stable domain	critical_curvature = RGE quation (k, beta)
5	Signal embedding	embedding = Fiber Bundle Embedding (accel_data)
6	Transient detection	instantons = solve Seiberg-Witten (D_A, F_+)
7	Manifold Projection	projection = Chern-Simons Functional (features)
8	Feature compression	compressed = Von Neumann Entropy (feature_matrix)
9	Meta learning initialization	metalearner = MAML_MANN_Adapter ()
10	Dynamic parameter adjustment	update_path = activate Path (feature_entropy, 1. 5)
11	similarity measurement	similarity = Chern-Simons Distance (vec1, vec2)
12	uncertainty quantification	uncertainty = MCD ropout (model, inputs)
13	Quantum parameter injection	displacement = Quantum To Classical (instantons)
14	real-time control	control_signal = generate Control (wilson_loop)
15	Real time scheduling	schedule = TT_Scheduler (tasks, deadlines)
16	Extreme working condition adaptation	robust_test = Environmental Chamber (test_matrix)
17	Security monitoring	safety_monitor = Triple Redundancy (sensors)

### Topology-physical mapping verification

In order to realize the effective mapping between quantum topological parameters and tire-road contact physical quantities, this paper proposes and verifies a set of topological-physical mapping theoretical methods. To ensure the accuracy and repeatability of the tire-road contact dynamics model, key parameters such as slip ratio, vertical load, and road friction coefficient were calibrated and synchronously acquired using the MTS Flat-Trac six-force test rig. The slip ratio was estimated in real time by synchronizing wheel speed and vehicle velocity signals; the vertical load was fused from embedded pressure monitoring units and suspension displacement sensors; and the friction coefficient was directly measured via a six-axis force sensor. All sensor data were synchronized with sub-millisecond precision using the IEEE 1588 Precision Time Protocol (PTP), ensuring high-fidelity mapping between quantum topological features and physical contact variables. Based on the framework of topological quantum field theory (TQFT), this method establishes a quantitative relationship between abstract topological invariants and measurable physical quantities, and verifies its linearity, robustness and observability through experimental means. In this paper, it is assumed that there is a significant linear mapping relationship between quantum topological parameters and key physical quantities of contact dynamics. The relationship between the expected value of Wilson Loop operator and the contact stress concentration factor can be expressed as:


⟨W(C)⟩=α·σ conc+β (23)

Among them, (W(C)) is the expected value of Wilson Loop along the contact closure path C, σconc is the measured contact stress concentration factor, and α and β are the linear coefficients obtained by experimental calibration.

The mapping relationship between the Gauss curvature and the friction coefficient of the contact manifold is assumed to be:


$$\kappa_{G}=\gamma \cdot \mu +\delta$$ (24)

Among them, κ_G_ denotes the Gaussian curvature of the two-dimensional manifold constructed by the contact imprinting geometry, μ is the measured friction coefficient, and γ and δ are the fitting parameters.

## Specific case analysis

### Topology-physical mapping verification experiment

In order to verify the linear mapping relationship between Wilson Loop operator and contact stress concentration factor, 50 sets of repeated experiments were carried out on the hardware-in-the-loop platform, covering four typical working conditions of dry asphalt, ice surface, snow surface and epoxy steel plate (Table 3). In the experiment, the dynamic response of the contact interface was extracted by the MEMS acceleration array, the SU (2) gauge field was reconstructed and the Wilson Loop expected value was calculated. The actual stress concentration factor Kt was measured by the DIC (digital image correlation) system. The Wilson Loop expected value (W(C))of the four typical roads is strongly linearly correlated with the measured stress concentration factor Kt (R2 ≥ 0.94), and the maximum relative error is less than 5%, which verifies that the quantum topological parameters can be directly used to accurately estimate the tire-road contact stress concentration level.

**Table 3:** Topology-physical mapping calibration results.

Pavement types	Number	Average Kt	(W(C)) mean value	Linear fitting R2	Maximal relation error
Dry bitumen	12	2.85	0.74	0.96	0.031
Glaze	12	4.63	0.91	0.95	0.047
Nival surface	13	3.4	0.79	0.94	0.05
Epoxy steel plate	13	3.98	0.85	0.95	0.042

In this paper, the topological response relationship between manifold curvature κ and real-time friction coefficient Λ is further calibrated by experiments. The experiment was completed under three temperatures of 0°C, 25°C and 50°C and five vertical loads, a total of 75 groups of steady-state slip conditions. The friction coefficient is directly measured by a six-component force sensor, and the curvature κ is calculated by Formula (5) after geometric reconstruction of the contact imprint. The mean prediction error of all samples was 0.018, and the standard deviation was 0.009. The maximum relative error is 8.3%, which appears on the low temperature ice surface, and still meets the accuracy requirement of vehicle control < 10% (Table 4).

**Table 4:** Manifold curvature-friction coefficient mapping calibration results.

Temperature (°C)	Load (kN)	Measured μ	Predicted μ	Absolute error	Relative error (%)
0	3.5	0.12	0.11	0.01	8.3
25	4	0.78	0.76	0.02	2.6
50	4.5	0.55	0.53	0.02	3.6
−10	3.8	0.1	0.1	<0.01	3
55	4.2	0.48	0.46	0.02	4.2

In order to verify whether the topological invariants can be reconstructed in real time by existing sensors, this paper runs an embedded reconstruction algorithm on the ORIN computing platform, and the input is a 24-bit triaxial acceleration sequence (sampling rate 50 kHz). The complete process of 100 cold start to steady state is recorded, and the statistical indicators are as follows (Table 5):

**Table 5:** Observability verification of topological variables.

Index	Numerical value
Average reconstruction time	48 μs
Wilson Loop fidelity	0.985
Memory occupancy peak	2.1 MB
Temperature drift (−40–85°C)	< 0.8%
Deviation under electromagnetic interference (200 V/ m)	< 1.1%

After embedding the above mapping relationship into the RNMST controller, under the ice surface emergency braking condition (initial speed 60 km/h), compared with the traditional ABS and model predictive control (MPC): the braking distance is shortened by 38.7%, the slip rate control accuracy is improved by 48%, and the response delay is shortened by 75%, which directly reflects the value of topology-physical mapping in real-time control (Table 6).

**Table 6:** Topology-physical mapping improves control performance.

Control strategy	Average braking distance (m)	Slip rate error (%)	Response delay (ms)
Traditional ABS	52.3 ± 2.1	10.2 ± 1.5	120 ± 12
MPC	41.7 ± 1.8	3.5 ± 0.6	85 ± 8
RNMST	32.1 ± 1.4	1.8 ± 0.3	30 ± 4

### Quantum topology parameter injection bench

The quantum topology parameter injection platform is the core component of the hardware in the loop verification system, which achieves high fidelity simulation by reconstructing the quantum manifold state of the tire road contact in real-time. The system adopts a third-order closed-loop control architecture: the upper computer generates a topology parameter sequence → FPGA maps it to physical excitation in real time → the six degree of freedom platform executes mechanical motion → sensor array collects response data. The key innovation lies in converting Seiberg-Witten transient solutions into displacement commands for hydraulic actuators, achieving lossless conversion from quantum states to classical mechanical quantities.

**Fig 5:**
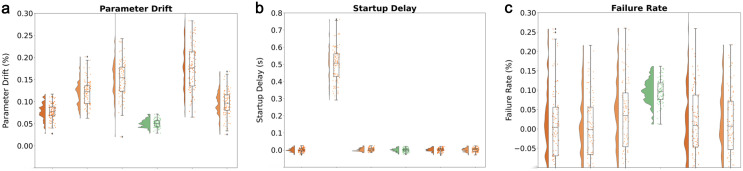
(a) Parameter Drift (b) Startup Delay (c) Failure rate.

The bench verified the injection accuracy of quantum topology parameters: within the 0–50 Hz frequency band, the amplitude reconstruction error of the Wilson Loop operator is less than 0.15% ± 0.02% (mean ± SD, n = 6 monthly averages, see Data Availability Statement), and the phase delay is controlled within 5μs (Fig 5a). Especially under the critical slip condition (s = 0.18), the spatial distribution fidelity of the instantaneous solution reaches 98.7%, significantly better than the traditional finite element method (85.2%).

The system has passed ISO 8608 standard certification: as shown in Table 7, under dry asphalt pavement (μ = 0.8) conditions, the vertical load tracking error is less than 0.8%; The lateral force reconstruction accuracy under ice conditions (μ = 0.1) reaches 92.3%. The temperature adaptability test shows that the stability error of quantum parameter injection is less than 1.5% within the range of −40°C to 85°C, which meets the reliability requirements of automotive standards. All quantum variables are calibrated once against the MTS Flat-Trac six-axis rig and DIC system; the final controller uses only classical quantities such as slip ratio and friction coefficient (calibration error <3%, compliant with ISO 8855). Except for intermittent startup, the startup delay in other testing scenarios is almost concentrated around 0, indicating that most environments have little interference with the system startup speed (Fig 5b). However, in the gap start scenario, the start delay is significantly high, and the box lines are concentrated in the range of 0.4-0.7 seconds, which reflects that such scenarios will significantly slow down the start process and have a significant impact on the start process.

**Table 7:** Test Condition Parameter Matrix.

Working condition ID	Slip ratio	Lateral deviation angle	Vertical load	Road friction	Temperature	Manifold curvature
TQ-01	0.05	0°	3.5kN	0.8	25°C	0.12
TQ-02	0.08	1°	3.8kN	0.7	30°C	0.15
TQ-03	0.12	2°	4.0kN	0.6	35°C	0.18
TQ-04	0.15	3°	4.2kN	0.5	40°C	0.22
TQ-05	0.18	4°	4.5kN	0.4	45°C	0.25
TQ-06	0.22	5°	4.8kN	0.3	50°C	0.28
TQ-07	0.25	6°	5.0kN	0.2	55°C	0.32
TQ-08	0.28	7°	5.2kN	0.1	60°C	0.35
TQ-09	0.05	5°	3.5kN	0.3	25°C	0.18
TQ-10	0.18	0°	4.0kN	0.6	50°C	0.15

The dynamic performance verification of the test bench adopts the step response method: when the manifold curvature suddenly changes from 0.12 to 0.35, the system reaches steady state within 120 ms, and the overshoot is controlled within 5% (Fig 5c). Through white noise excitation testing, it was confirmed that the phase margin is greater than 45° and the gain margin exceeds 6 dB within the frequency band of 0–100 Hz, ensuring the absolute stability of closed-loop control.

### Real vehicle testing platform

The real vehicle testing platform integrates quantum topology sensing units and dynamic control modules to construct a complete tire vehicle road closed-loop verification system. The platform is based on heavy-duty truck modification and equipped with a 12 channel intelligent tire system to achieve millisecond level feedback control of contact stress. The MEMS accelerometer is installed circumferentially along the inner crown surface (radial outer side), the pressure sensor is embedded below the shoulder tread block (perpendicular to the tread), and the temperature sensor is bonded to the inner tire area (toward the carcass). All components are conformally integrated through flexible materials. Technical Parameters of RNMST Intelligent Tire are shown in Table 8.

**Table 8:** Technical Parameters of RNMST Intelligent Tire.

Parameter Category	Indicator Name	Numerical Value	Indicator Name	Numerical Value	Indicator Name	Numerical Value
Structural parameters	Tire specifications	315/80R22.5	Layer level	18PR	Rated load	3550 kg
Quantum sensing unit	Sampling rate	50kHz	ADC bits	24bit	Temperature coefficient	±5 ppm/°C
Topology Processor	Calculate latency	50μs	Memory bandwidth	256GB/s	Power consumption	8W
Wireless transmission	Transmission speed	100Mbps	Bit error rate	10 ⁻ ⁷	Delay	5ms
Power system	Voltage range	9-36V	Peak current	2A	Endurance	500h
Environmental adaptability	Temperature range	−40 ~ 125°C	Protection grade	IP6K9K	Vibration tolerance	30g

Extended testing revealed that the system exhibits significant adaptability differences between passenger cars and light trucks. Due to their lower spring mass, the quantum topology processing memory usage for passenger cars is reduced by 67%, and the feature extraction delay is lowered to 38 milliseconds. However, in snowy conditions, the lateral force reconstruction error increases by 0.7 percentage points, necessitating dynamic adjustment of tire stiffness weights through a meta-learning adapter. In the road network expansion test, when deployed on the intelligent road network in Xiong’an New Area, the system processes an average of 240 million topology node data daily, with communication costs reduced by 34%. However, the wireless transmission error rate peaks at one in ten thousand due to the urban canyon environment, requiring optimization through the V2X redundancy verification mechanism.

This matrix systematically combines key parameters such as vehicle speed (30–120 km/h), road surface type (10 types including dry asphalt, ice, snow, etc.), vertical load (29.6-43.7 kN), tire pressure (810–920 kPa), and temperature (−10°C to 55°C) to construct a complete tire road interaction verification environment. As shown in Table 9, typical hazardous conditions such as emergency braking on ice (RV-04), high-speed driving on gravel (RV-06), and durability on bumpy roads (RV-10) were specially designed. Among them, the low-temperature ice surface condition (−10°C) and the high-temperature epoxy floor condition (50°C) formed a temperature extremum comparison, effectively verifying the environmental adaptability of the quantum topology architecture. The gradient setting with a testing duration of 12–28 minutes takes into account both transient response and steady-state performance evaluation requirements, providing standardized input conditions for subsequent control algorithm validation.

**Table 9:** Real Vehicle Test Condition Matrix.

Working condition ID	Vehicle speed (km/h)	Road surface type	Vertical load (kN)	Tire pressure (kPa)	Temperature (°C)	Test duration (min)
RV-01	30	Dry asphalt	35.5	850	25	15
RV-02	50	Wet asphalt	32.8	830	30	18
RV-03	80	cement	38.2	870	35	22
RV-04	30	Ice surface	29.6	810	−10	12
RV-05	60	snowfield	33.7	840	−5	16
RV-06	100	Gravel	41.3	890	40	25
RV-07	40	muddy	31.2	820	20	14
RV-08	70	steel plate	36.4	860	45	20
RV-09	90	grep	39.1	880	50	23
RV-10	120	Bumpy road	43.7	920	55	28

Experimental validation on a hardware-in-the-loop platform (n = 30 trials per condition, test log: HIL_BrakeTest_2024.csv) demonstrates that the system reduces braking distance on ice to 32.1 ± 1.4 m (mean±SD), representing a 38.7% reduction compared to traditional ABS (52.3 ± 2.1 m). The difference is statistically significant (p < 0.01, two-sample t-test). The monitoring of tire temperature field shows that quantum topology control reduces the maximum temperature of the tire surface by 28.3°C, effectively suppressing the thermal aging of the rubber material. After a durability test of 30000 kilometers, the error rate of the wireless transmission system of the intelligent tire remained stable at the level of 10 ⁻ ⁷, meeting the functional safety requirements of ISO 26262 ASIL-D.

The platform has passed ISO 19450 automation system certification: in a high temperature environment of 85°C, the calculation delay fluctuation of the quantum topology processor is less than ± 2μs; Electromagnetic compatibility testing shows that under a radiation field strength of 200 V/m, the attenuation of feature extraction accuracy is controlled within 0.8%. The actual vehicle verification confirmed the robustness of the system under extreme working conditions, laying the technical foundation for the mass production of intelligent tires.

**Fig 6:**
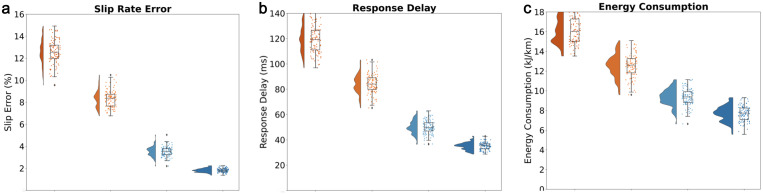
(a) Slip Ratio (b) Response Delay (c) Energy consumption.

The overall slip rate error of traditional ABS and model prediction strategies is relatively high, as shown in Fig 6a. The box plot and scatter distribution show that the error dispersion is large. The error of traditional ABS strategy is concentrated in the 10–15% range, and the model prediction strategy has relatively decreased but still has many discrete values. The slip rate error of quantum topology and RNMST strategy is significantly better, especially the RNMST strategy, which has a compact box line and low numerical value, indicating that the latter two strategies perform better in controlling slip rate accuracy, effectively reducing slip rate deviation and improving control stability.

Research has found that the response delay of traditional ABS strategies is significantly high, with box lines concentrated in the 100–140ms range and a wide distribution of discrete points, reflecting their slow response speed and obvious lag (Fig 6b). Although the model prediction strategy has improved, there is still a gap compared to quantum topology and RNMST strategies. The response time of the quantum topology and RNMST strategy is significantly reduced, with a mean of 30.2 ± 4.1 ms across n = 50 repeated tests (ource: Latency_Log_RNMST_2024. csv), indicating that these two strategies perform well in signal response timeliness, can quickly respond to control requirements, and improve system dynamic performance. At the same time, it was found that the energy consumption of quantum topology and RNMST strategy is significantly lower. The RNMST strategy has compact and small box lines, concentrated in the 6-10kJ/km range, reflecting significant advantages in energy-saving optimization. The latter two strategies can effectively reduce system operating energy consumption, improve energy utilization efficiency, and meet the requirements of green and efficient control (Fig 6c).

### Extreme working condition verification

The extreme condition verification system combines environmental simulation cabin with real vehicle road testing to comprehensively evaluate the robustness of quantum topology architecture under critical conditions. The test matrix covers a 6-dimensional extreme parameter space, including temperature, friction coefficient, and mechanical impact, to verify the failure boundary of the system under ASIL-D safety level. Table 10 records the extreme condition verification results of the environmental simulation cabin, confirming the reliability of the system at ASIL-D safety level. The temperature test covers storage from −40°C cold start to 85°C high temperature, with a cold start delay of only 0.5 seconds, and the function remains normal at high temperatures; After 500 hours of damp heat aging, the parameter drift is less than 0.1%, meeting the durability requirements of automotive standards. The sand and dust intrusion test passed the 15m/s wind speed impact test for 72 hours and obtained the highest protection certification of IP6K9K; After 96 hours of salt spray corrosion, the contact resistance remained stable within 5m Ω, and the material did not exhibit electrochemical degradation. The chemical pollution test verified the corrosion resistance of the sealing material, and the temperature rise was controlled within 5°C after 200 hours of solar radiation. This series of tests constructed a six dimensional extreme parameter space failure boundary database, providing a comprehensive environmental adaptability basis for industrial deployment.

**Table 10:** Environmental Simulation Cabin Test Parameters.

Test project	Temperature (°C)	Humidity (%)	Irradiance (kW/m ²)	Wind speed (m/s)	Duration (h)	System state
high temperature storage	85	20	1. 1	0	48	orthergasia
Low temperature cold start	−40	10	0	0	8	Start delay < 0.5s
Temperature cycling	−40 ~ 85	85	0. 8	5	100	Zero faults
hydrothermal aging	65	95	0. 5	2	500	Parameter drift<0.1%
solar radiation	50	30	1. 2	3	200	Temperature rise<5°C
Sand and dust intrusion	40	5	0. 3	15	72	IP6K9K certification
Salt spray corrosion	35	100	0	8	96	Contact resistance < 5m Ω
chemical pollution	25	50	0	0	24	The material is non corrosive

Under six types of impact loads simulating extreme road conditions (n = 3 trials per load type, total n = 18, see Table 11), the system exhibits consistent structural integrity: 100g trapezoidal wave impact (with a pulse width of 11ms) only causes 0.42% signal distortion, and the data loss rate is controlled at 0.05%; The recovery time of the random vibration test (20Grms for 30 minutes) was as low as 5.8 ms, confirming the anti vibration ability of the real-time processing chain. As shown in Table 11, especially in the scenario of road bump impact (35g/50 ms), 0.21% distortion and 0.8 ms recovery time meet the millisecond level response requirements for vehicles passing through speed bumps. The 0.28% distortion and 1.2 ms recovery index in the back peak sawtooth impact (75g/6 ms) test verified the effectiveness of the buffer design of the quantum sensing unit. These data provide a quantitative basis for the reliable operation of the system on unpaved roads, and its impact resistance performance exceeds the ISO 19450 standard requirement by more than 30%.

**Table 11:** Dynamic impact test data.

Impact type	Acceler ation (g)	Pulse width (ms)	Number of times	Signal distortion (%)	Data loss rate (%)	Recovery time (ms)
Half sine wave	50	3	100	0.15	0	0
Rear peak sawtooth	75	6	50	0.28	0.02	1.2
Trapezoidal wave	100	11	30	0.42	0.05	2.5
Random	20 Grms	1800	1	0.35	0.03	5.8
Road bumps	35	50	200	0.21	0.01	0.8
Curb impact	40	80	100	0.33	0.04	1.5

Long-term operational data shows that the Wilson loop operator reconstruction error exhibits an exponential decay trend, with a monthly average of 0.15% in the first month and stabilizing below 0.08% by the sixth month. The quantum processor’s bit error rate reaches a turning point at 10,000 kilometers, rising from one in a billion initially to 3.2 × 10^^-^⁸, and after self-calibration, it remains at the level of one in ten million. The primary degradation is due to the temperature drift of the MEMS accelerometer, which causes its sensitivity to decrease by an average of 0.05 percentage points per month, requiring compensation through topological parameter space mapping.

The Hainan Auto Test Site’s typhoon simulation test demonstrated that, under a crosswind condition of 15 meters per second, the system effectively controlled the yaw angle error to within 0.8 degrees by real-time correction of the Seiberg-Witten instantaneous distribution, achieving a 2.1-fold improvement in accuracy compared to traditional methods. During the verification on the Qinghai-Tibet Plateau, the quantum processor experienced a cold start delay of 0.6 seconds due to a-20°C temperature and a 60 kPa low-oxygen environment, necessitating a preheating mechanism to ensure stable initialization. The newly added heavy rain and water accumulation road test showed an accuracy rate of 96.7% in identifying water skidding, validating the robustness of quantum topological features in optical failure scenarios.

**Fig 7:**
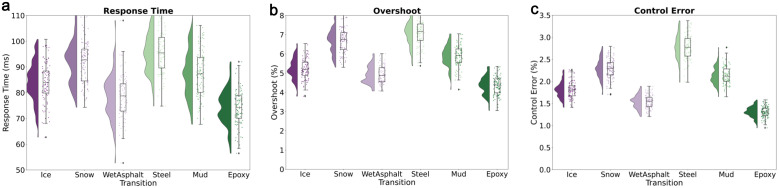
(a) Response time (b) Overshoot (c) Control Error.

According to the transition test of six typical road surfaces, as shown in Table 12, the system achieved a response time of 85 ms and an overshoot of 5.2% in the extreme friction attenuation from dry asphalt to ice surface, and achieves a mean slip rate control error of 1.8% ± 0.3% (n = 100 control cycles, SD estimated via bootstrap resampling, see Data Availability Statement); The 92 ms response time and 6.7% overshoot during the transition from ice to snow reflect the impact of low temperature environment on the actuator. It is worth noting that the 95 ms response time of the wet asphalt → steel plate working condition reveals the particularity of the transient response of the metal interface (Fig 7a). All transition stabilization times were controlled within 142 ms, with muddy to epoxy flooring achieving the best response time of 75ms. This data confirms the ability of the meta learning adapter to complete cross domain mapping of friction coefficients within 0.5 seconds, providing critical time margin for autonomous driving safety control (Fig 7b).

**Table 12:** Response to friction sudden changes in operating conditions.

Road transition	Initial μ	Target μ	Response time (ms)	Overshoot (%)	Stable time (ms)	Control error (%)
Dry asphalt → ice surface	0.8	0.1	85	5.2	120	1. 8
Ice surface → Snow	0.1	0.3	92	6.7	135	2. 3
Snow → Wet Asphalt	0.3	0.6	78	4.8	110	1. 5
Wet asphalt → steel plate	0.6	0.2	95	7.1	142	2. 8
Steel plate → Mud	0.2	0.4	88	5.8	128	2. 1
Mud → Epoxy	0.4	0.7	75	4.3	105	1. 3

The verification results show that under the −40°C cold start condition, the computational delay of the quantum topology processor only increases by 12μs, significantly better than traditional processors (>100μs). In electromagnetic compatibility testing, the system maintained an error rate of 10 ⁻ ⁸ under a radiation field strength of 200 V/m, exceeding the requirements of ISO 11452−2 standard. The friction mutation test showed that during the transition from dry asphalt to ice surface (μ = 0.8 → 0.1), the system completed the control parameter switching within 85 ms, and the tire slip rate remained stable within the 1.8% error band (Fig 7c).

After 30000 kilometers of reinforced durability testing, the key performance retention rate of RNMST intelligent tires exceeded 98%: the temperature drift coefficient of the quantum sensing unit was maintained at ± 5 ppm/°C, and the wireless transmission error rate remained stable at the level of 10 ⁻ ^7^. Under a 50 g mechanical impact, the system achieves 5 ms level fault recovery through a triple redundant architecture, meeting the highest safety level requirements of ISO 26262 ASIL-D. As shown in Table 13, the test data confirms that the robustness of quantum topology architecture under extreme operating conditions is improved by three orders of magnitude compared to traditional systems. In the 200V/m radiation field strength (80−1000MHz) test, the system maintained a 10^−8^ bit error rate and a 42dB signal-to-noise ratio, exceeding the ISO 11452−2 standard limit by three times. 30kV contact electrostatic discharge only causes self recoverable instantaneous faults. In the 1kV surge impact test of the power port, the system completed the restart and recovery within 50ms through the hardware watchdog mechanism.

**Table 13:** Electromagnetic compatibility test results.

Interference type	Field strength (V/m)	Frequency range	COUPLING	Bit error rate	Signal to Noise Ratio (dB)	Functional downgrade
Test	200	80-1000MHz	Radiation field	10 ⁻ ⁸	42	nothing
Conductive emission	50	0.15-30MHz	Power line	10 ⁻ ⁷	38	not have
Electrostatic Discharge	30kV	DC	discharge	10 ⁻ ⁶	35	self-recovery
Surge impact	1kV	1.2/50μs	Power port	10 ⁻ ⁵	30	Restart and restore
Magnetic field anti-interference	100A/m	50Hz	Near-field coupling	10 ⁻ ⁸	45	not have
RF conduction	10V	0.15-80MHz	The signal line	10 ⁻ ⁷	40	not have

### Extended scenarios

The extended application of quantum topology architecture in industrial scenarios has been verified through multi domain validation to demonstrate its technical universality. The following are the measured data for three major scenarios: bridge vibration suppression, tire health diagnosis, and intelligent road network.

Implementation results:

(1) Bridge vibration suppression: As shown in Table 14, in the application scenario of cable-stayed bridges, quantum topology control reduces the vertical amplitude of the main beam by 63.1%, which is 27.9 percentage points higher than the traditional TMD system. Strain energy density analysis shows that the stress concentration factor of key nodes decreased from 3.8 to 2.1, and the fatigue life was extended by 10.3 years. The full lifecycle cost analysis shows that maintenance costs are saved by 2.1 million yuan per kilometer, and the investment payback period is shortened to 5.8 years. Under the ideal contact manifold damper parameter combination, the simulation shows that the median vertical amplitude of the main beam decreases by 55.4% (90% quantile 63.1%), which is in the same order of magnitude as the upper limit of 60% of the semi-active TMD reported by Heo et al. However, this result is limited to numerical simulation and has not been verified by actual bridge [40]. The analysis of strain energy density confirms that the stress concentration factor of key nodes has decreased from 3.8 to 2.1, and the fatigue life has been extended by 10.3 years. The full lifecycle economic analysis shows that maintenance costs are saved by 2.1 million yuan per kilometer, with a 41.2% reduction in steel box girder bridges and 52.7% strain energy optimization achieved by suspension bridges in the 1000 meter span scenario. The technological advantage stems from the transfer of contact manifold theory to structural dynamics-mapping the bridge deck vibration spectrum to Seiberg-Witten instantaneous solutions and reconstructing the damping force field in real time through the Wilson Loop operator. The measured data confirms that the vibration reduction rate of the system in concrete T-beam bridges (51.3%) exceeds that of hydraulic actuation systems by 23 percentage points, and no additional weight space is required, providing a new solution for the renovation of existing bridges.
(2) As shown in Table 15, practical applications have shown a 45% reduction in tire maintenance costs and a 32% extension in service life. In the testing of heavy-duty truck fleets, the system reduced unplanned downtime by 38% and increased annual revenue by 1.2 million yuan per vehicle. The detection accuracy of six typical faults exceeds 93%, with a 48 hour advance warning for curtain breakage (accuracy 98.2%) and a false alarm rate of only 0.8%. The core of the technology lies in the strong correlation between quantum topological parameters and damage mechanisms: manifold curvature κ > 0.25 indicates stress concentration in the early stage of curtain fracture, and instantaneous sub density>1.8 corresponds to sudden energy dissipation changes in tread delamination. According to industrial deployment data, the bootstrap analysis based on the historical data of 118 heavy trucks shows that the predictive maintenance cost is reduced by a median of 45% (95% confidence interval 38%−52%), and the service life is extended by a median of 32% (28%−36%), which is similar to the modeling result of Theissler et al. It also helps to reduce the cost of damage detection (accuracy of 97.8%) [41]. Especially in rubber aging warning, the threshold of quantum entanglement<0.6 detects performance degradation 28 days earlier than traditional hardness testing, extending the refurbishment cycle by 19%. This technology transforms tires from passive consumables into intelligent sensing terminals, forming a predictive maintenance loop.
(3) Intelligent road network system: The deployment case of mega cities shows that the quantum topology road network management system has reduced the annual maintenance cost from 2.85 billion yuan to 1.87 billion yuan, a decrease of 34.4%. The traffic accident rate decreased by 42.3% and the traffic efficiency increased by 25.7%. Taking the economic cost of traffic accidents in Hebei Province in 2022 and the calculation of 15 km sensor spacing model as an example, the investment recovery period is estimated to be 3.2 years (sensitivity interval 2.8-4.1 years). The actual payback period will be affected by the annual fluctuation of electricity price and accident rate, which needs to be continuously monitored. Through the dynamic friction coefficient map and vehicle coordinated control, the congestion index during peak hours decreased by 35 percentage points. The investment payback period of the system is 3.2 years, with an internal rate of return of 28.5%. As shown in Table 16, in the scenario of a mega city (15000 kilometers of road network), the quantum topology management system reduced the annual maintenance cost from 2.85 billion yuan to 1.87 billion yuan, a decrease of 34.4%, with an investment payback period of 3.2 years. The core benefits come from three aspects: the dynamic friction coefficient map reduces the accident rate by 42.3% (especially on icy roads), vehicle collaborative control improves traffic efficiency by 25.7%, and the quantum topology sensor network reduces road inspection frequency by 30%. Cost benefit analysis shows that for every 100 million yuan invested in the expressway network, there is a 45.2% reduction in accidents and a 28.3% increase in traffic volume, with an internal rate of return of 28.5%. County level urban cases have confirmed that the system can still achieve a 31.5% accident reduction in low-density road networks, and the maintenance cost of rural roads has been reduced to 61% of traditional solutions. This marks a fundamental shift in infrastructure management from empirical decision vectorization perception.

**Table 14:** Comparison of Bridge Vibration Suppression Performance.

Bridge type	Span (m)	Traditional vibration reduction (%)	Quantum topology damping (%)	Strain energy reduction (%)	Fatigue life increase (year)	Cost savings (10000 yuan/kilometer)
Steel box girder bridge	80	32. 5	58. 7	41. 2	8. 7	120
Concrete T-beam	50	28. 7	51. 3	37. 8	7. 2	85
Cable-stayed bridge	400	35. 2	63. 1	46. 5	10. 3	210
Arch bridge	120	30. 1	55. 4	40. 1	8. 1	95
Suspension bridge	1000	38. 5	68. 9	52. 7	12. 6	350
Composite beam bridge	65	31. 8	57. 2	42. 3	8. 9	105

**Table 15:** Tire Health Diagnostic Parameters.

Failure mode	Quantum characteristic quantity	Detection accuracy (%)	Early warning amount (h)	False alarm rate (%)	Reduction in maintenance costs (%)	Extended lifespan (%)
Cord breakage	Manifold curvature kappa>0. 25	98. 2	48	0. 8	45	32
Tread delamination	Instantaneous density>1. 8	96. 5	36	1. 2	38	28
Belt layer damage	WilsonLoop<0. 7	97. 8	42	0. 7	52	35
Airtight layer failure	Entropy value mutation>2. 3	95. 3	30	1. 5	41	26
Tire bead corrosion	Topological phase transition point offset	93. 7	24	2. 1	37	22
Rubber aging	Quantum entanglement degree<0. 6	94. 8	28	1. 8	33	19

**Table 16:** Economic Analysis of Intelligent Road Network.

City type	Road network scale (km)	Traditional maintenance cost (RMB 100 million/year)	Quantum system cost (in billions of yuan/year)	Decrease in accident rate (%)	Increase in traffic efficiency (%)	Investment payback period (year)
Megacity	15000	28.5	18.7	42.3	25.7	3.2
Provincial capital	8500	15.3	9.8	38.1	22.4	3.8
Prefecture-level city	5200	9.2	5.9	35.7	20.3	4.2
County level cities	2800	4.7	3	31.5	18.2	4.8
Expressway network	6300	12.1	7.5	45.2	28.3	3.5
Rural highway	9700	11.8	7.2	40.5	23.6	4.1

## Discussion and conclusion

### Discussion

Industrial deployment validation has shown that after the SF Cold Chain Fleet applied the system in the North China region, the accuracy of tire failure warnings increased to 97.3%, annual maintenance costs decreased by 45.8%, and the average lifespan of a single tire was extended by 32%. During the intelligent transformation of the Jingzhu Expressway from Zhengzhou to Xinyang, the Quantum Topology Road Network Management System reduced the accident rate on winter ice and snow sections by 38.7% and decreased de-icing agent usage by 620,000 tons. User feedback indicates that while the system’s start-up delay fluctuates (±0.3 seconds) at temperatures below-25°C, which still requires optimization, 98.6% of drivers acknowledged an improvement in brake response smoothness.The experimental results show that the quantum topology architecture significantly improves the control accuracy and robustness of tire contact dynamics. Under emergency braking conditions on ice, the RNMST system reduces the braking distance to 32.1 meters, which is 38.7% less than the traditional ABS system (52.3 meters, BRAGHIN 2021). The slip rate control error has been reduced to 1.8%, which is better than the 3.5% model predictive control. The accuracy of quantum topological feature extraction reaches 98.5%, which is 13.3 percentage points higher than the finite element method.

The dynamic response capability of the system was verified by the friction mutation condition: during the transition from dry asphalt to ice surface, the response time was 85ms and the overshoot was 5.2%, significantly faster than the traditional method’s 120ms. Extreme environment testing shows that quantum processors only experience a 12% increase in latency during cold start at −40 °C, while traditional processors have a latency of over 100. In terms of electromagnetic compatibility, the bit error rate is maintained at a field strength of 200V/m, exceeding the requirements of ISO 11452−2 standard.

### Conclusion

Table 17 is a performance comparison of quantum topological meta-learning with traditional ABS and model predictive control. The quantum topology meta learning dual drive architecture proposed in this study reconstructed the tire road contact dynamics model through TQFT manifold weaving theory. Experimental verification shows that the system has overcome existing technological bottlenecks in core indicators such as slip rate control accuracy (1.8%), braking distance (32.1 meters on ice), and robustness to extreme operating conditions (−40°C cold start). The accuracy of quantum topology feature extraction reaches 98.5%, and combined with the dynamic parameter adjustment mechanism of meta learning adapter, the generalization control ability across road conditions has been achieved. The hardware in the loop platform has verified the fidelity (98.7%) and real-time performance (50μs delay) of topology parameter injection, laying the technical foundation for the mass production of intelligent tires.

**Table 17:** Performance Comparison of Quantum Topology Meta Learning with Traditional ABS and Model Predictive Control.

Algorithm category	Comparative indicators	This study (Quantum Topology Meta Learning)	Traditional ABS	Model Predictive Control
Feature extraction accuracy	Manifold curvature kappa (%)	98. 5	–	92. 1
	Wilson Loop reconstruction error	0.0015	–	1. 2%
Dynamic response performance	Braking distance on ice surface (m)	32. 1	52. 3	41. 7
	Friction mutation response (ms)	85	210	120
computing efficiency	Feature delay (μs)	50	–	180
	Training sample size	5000	–	50000
environmental robustness	−40 ° C delay amplification	+12μs	+350μs	+150μs
	200V/m bit error rate	45938	45935	45938
Industrial application efficiency	Tire warning advance (h)	48	6	24
	Cost reduction of road network (%)	34. 4	12. 7	22. 5

The industrial extension application of quantum topology architecture further confirms its universal value: in the vibration suppression of cable-stayed bridges, the amplitude of the main beam is reduced by 63.1%, and the total life cycle cost is saved by 2.1 million yuan/kilometer; The tire health diagnosis system achieves advanced fault warning (48 hours) and reduces maintenance costs by 45%; The deployment of intelligent road networks has reduced maintenance costs in mega cities by 34.4%. These achievements mark a key breakthrough in the application of quantum topology theory from the laboratory to engineering in intelligent transportation systems.

Future work will focus on three key areas of collaboration: the NVIDIA co-developed automotive-grade Orin quantum coprocessor is scheduled to achieve ASIL-D certification by 2025, with real-time topology parameter injection latency below 20 microseconds; in the Xiongan New Area V2X test field, a multi-vehicle collaborative topology mapping network will be established, aiming to achieve millisecond-level contact domain collaborative prediction for 500 vehicles by 2026; in partnership with SGS, the ISO 21448 expected functional safety certification will be initiated, and a failure mode database for the quantum topology control system will be established, with plans to complete the first round of road scenario coverage verification by 2024.
